# Facile Synthesis of Oxazolidinones as Potential Antibacterial Agents

**DOI:** 10.1002/open.202400432

**Published:** 2025-01-07

**Authors:** Secret P. Els, Kimberleigh B. Govender, Mxolisi K. Sokhela, Nilay Bhatt, Nakita Reddy, Hendrik G. Kruger, Per I. Arvidsson, Hendra Gunosewoyo, Thavendran Govender, Tricia Naicker

**Affiliations:** ^1^ Discipline of Pharmaceutical Sciences Catalysis and Peptide Research Unit University of KwaZulu-Natal Durban 4000 South Africa; ^2^ Science for Life Laboratory Drug Discovery & Development Platform and Division of Translational Medicine and Chemical Biology Department of Medical Biochemistry and Biophysics Karolinska Institutet Stockholm Sweden; ^3^ Curtin Medical School, Faculty of Health Sciences Curtin University Bentley, Perth WA 6102 Australia; ^4^ Department of Chemistry University of Zululand, Private Bag X1001 KwaDlangezwa 3886 South Africa

## Abstract

An efficient microwave‐assisted synthesis route for novel oxazolidinone analogues has been developed. The general synthesis of these compounds began with an L‐proline‐mediated three‐component Mannich reaction between commercially available 3‐fluoro‐4‐morpholinoaniline, aqueous formaldehyde and α‐hydroxyacetone. This was followed by a one‐step cyclisation to form the core structure of oxazolidinone antibiotics which was subsequently derivatized. The novel compounds were evaluated for their antibacterial activity against *M. smegmatis*. One of the novel oxazolidinone derivatives **18 a1** produced a MIC of 8 mg/L, comparable with the commercial Rifampicin. The methodology is a useful addition to the field since it can make highly sought‐after oxazolidinone derivatives, using cheaper, less harsh commercially available reagents, in a short time and one pot.

## Graphical abstract

A microwave‐assisted method synthesizes novel oxazolidinone analogues via a proline‐initiated three‐component Mannich reaction and subsequent cyclization. The resulting analogues show antibacterial activity against *M. smegmatis*, with a minimum MIC of 8 mg/L, which is comparable to Rifampicin. This approach offers a valuable, efficient route to oxazolidinone derivatives with readily available reagents.

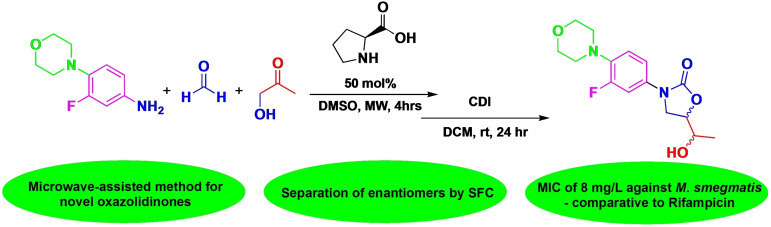

## 1 Introduction

In 2021, the global death toll from Tuberculosis (TB) reached roughly 10.6 million recorded cases and 1.6 million individuals, encompassing both TB‐related deaths and those involving HIV, which accounted for 187,000 cases.[^1^, ^2^] TB stands as the thirteenth primary contributor to global mortality and the second most prominent infectious cause of death, surpassing even HIV and AIDS, with only COVID‐19 ranking higher. The challenge of multidrug‐resistant TB (MDR‐TB) persists as a concern for public health and health security. Merely a third of individuals affected by drug‐resistant TB were able to obtain treatment in the year 2021. The most commonly used antibiotics such as Isoniazid, Rifampicin, Ethambutol, Pyrazinamide, Moxifloxacin, and Bedaquiline are failing to overcome drug resistance which continues to be a public health threat.[^3^, ^4^, ^5^]

**Figure 1:**
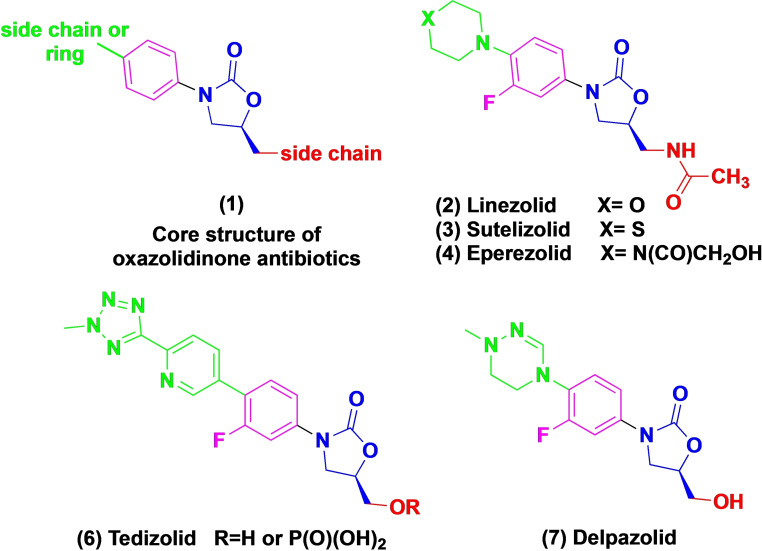
Some representative oxazolidine analogues licensed or under development.^[10].^

The discovery of oxazolidinone compounds (characterized by a basic nucleus of 2‐oxazolidone) brings hope to the fight against the MDR challenge.[^6^, ^7^, ^8^, ^9^, ^10^, ^11^] Oxazolidinones are bacteriostatic agents derived synthetically with no congeners found in natural products. These compounds were first reported in 1978, and today are well known for their antibacterial properties against gram‐positive bacteria such as methicillin‐resistant *Staphylococcus aureus* (MRSA) and vancomycin‐resistant enterococci (VRE).[^6^, ^7^, ^9^, ^12^, ^13^, ^14^, ^15^, ^16^] Currently, several derivatives of oxazolidinones are either in development or already approved for clinical use to treat TB or other bacterial infections (Figure 1).[^10^, ^12^, ^17^] Linezolid and Tedizolid are clinically approved for MDR‐TB infections.^[10]^ Linezolid is widely employed for Gram‐positive bacterial infections and it is considered an efficient drug for surgical infections.^[11]^ Tedizolid phosphate was the second oxazolidinone drug approved by the FDA for treating MRSA skin infections.[^18^, ^19^]

Sutezolid and Delpazolid are progressing into Phase 2b regimen trials involving drug‐resistant TB patients.[^20^, ^21^] These trials focus on achieving shortened treatment durations and, if proven effective, these drugs might also find utility in managing MDR‐TB. The insights gained from these trials will enhance our comprehension of the potential contributions of oxazolidinones in reducing treatment durations, building upon observations from mouse models. Moreover, this could expedite the selection and development of novel, safer oxazolidinone compounds.

The recent ZeNix clinical trials involving a treatment regimen of Bedaquiline‐Pretomanid‐Linezolid revealed a groundbreaking achievement: the potential to successfully cure most patients with drug‐resistant TB within 6 months. The ongoing Phase 3 trials incorporating these medications hold the promise of refining and advancing optimized treatment regimens even further.^[22]^

Oxazolidinone compounds possess a unique mode of action, due to their broad spectrum activity against MDR gram‐positive and anaerobic pathogens.[^7^, ^9^, ^12^, ^15^] This class of compounds inhibits the protein synthesis of bacteria at a very early stage, by binding selectively to the central loop of the 23S rRNA domain of the 50S ribosomal subunit.^[7]^ This mode of action reduces the chance of cross‐resistance with other antimicrobial agents.^[7]^

To overcome multi‐drug resistance, improve antibacterial efficacy and avoid long‐term side effects, there is still a large driving force for developing new derivatives based on the oxazolidinone scaffold.[^15^, ^23^, ^24^] The emergence of Linezolid‐resistant bacteria has raised safety concerns due to the drug's interference with mitochondrial protein synthesis.^[25]^ As a result, researchers have begun investigating Linezolid analogues and oxazolidinone derivatives to increase their antibacterial spectrum.[^10^, ^23^, ^26^, ^27^, ^28^, ^29^, ^30^]

Although the high price of anti‐TB medication mainly reflects a long and expensive development cycle, for example, in the case of Linezolid (9 synthetic steps), the market price also reflects a limitation of the current processes involved in its synthesis.^[31]^

The improved syntheses of typically sought‐after oxazolidines (Linezolid, Tedizolid, Sutezolid and Delpazolid etc.) still require at least 6 steps that involve, expensive reagents, complex condensation and cyclization reactions (such as lithiations).[^32^, ^33^, ^34^, ^35^, ^36^, ^37^, ^38^] The commercial unavailability of several building blocks has led to increased steps during syntheses. Hence, in addition to discovering more oxazolidinone analogues, it is necessary to develop more concise and cost‐effective synthetic routes, that are better suited to the large‐scale production of these drugs.

Organocatalyzed three‐component direct Mannich reactions are one of the types of transformations that are useful in asymmetric synthesis, more especially in the construction of nitrogen‐containing chiral organic compounds.[^39^, ^40^, ^41^] These reactions take place between aldehydes, ketones and amines in the presence of an organocatalyst to yield the corresponding β‐amino ketones.^[42]^ Most of the reported Mannich reactions, are proline‐catalyzed and represent good to excellent yields and stereoselectivities.[^40^, ^41^, ^42^, ^43^] Our group has reported using this methodology for the synthesis of β‐lactam carbapenem intermediates.^[44]^

To the best of our knowledge, proline‐mediated three‐component Mannich reactions have not been utilized in the synthesis of oxazolidinone derivatives. Herein, we report the synthesis of these compounds via a microwave‐assisted proline‐initiated direct Mannich reaction as a key step. In addition, all products were evaluated for bacterial activity against *Mycobacterium smegmatis* PJV 53.

## 2 Results and Discussion

Even though various organocatalyzed approaches have been developed to improve direct Mannich reactions, only a few methods reported using formaldehyde as the reacting aldehyde.[^44^, ^45^, ^46^, ^47^] Given the known difficulties with imine formation using formaldehyde for the Mannich reaction, we initiated our study following those procedures successful with this reagent, as reported by Córdova,^[46]^ Bolm^[45]^ and Govender.^[44]^

The reactions between 3‐fluoro‐4‐morpholinoaniline (**8**), aqueous formaldehyde (**9**), and α‐hydroxyacetone (**10**), using 30 mol % of L‐proline (**12)** as a catalyst in DMSO gave conversions of around 20 % of starting material **8** and trace amounts of the Mannich product **11** as observed by LC–MS. Córdova *et al*.^[46]^ reported a similar proline‐catalyzed reaction using 4‐methoxyaniline instead of 3‐fluoro‐4‐morpholinoaniline and was able to drive the reaction to completion in 17 hours with a good yield (60 %).^[46]^ Thus, our results are in line with a report by Liang,^[47]^ which concluded that anilines with electron‐withdrawing substituents tend to show lower reactivities with formaldehyde.[^47^, ^48^]

**Scheme 1:**
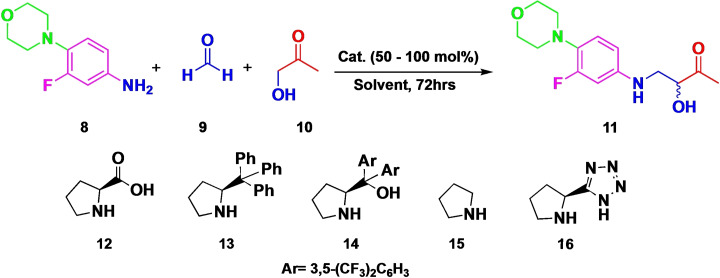
Organocatalyzed direct Mannich reaction.

After these initial disappointing results, various experimental conditions were employed to improve the transformations. Based on the initial results, we then tried different experimental conditions to improve the transformation (Scheme 1). Increasing the catalyst to 50 mol % resulted in 20 % conversion after 72 hours, with approximately half of the product being the desired compound and the remainder consisting of unidentified side products (presumably aldol and imine adducts). Common amino catalysts **13** and **14** demonstrated significantly lower efficiency, with conversions dropping to as low as 5 %. Despite increasing the catalyst loading, the conversion could not be increased. Subsequently, we employed a non‐chiral pyrrole (**15**), which proved ineffective, suggesting that a catalyst with a free carboxylic acid moiety is essential for the reaction to proceed. We then tested the tetrazole derivative (*S*)‐(−)‐5‐(2‐pyrrolidinyl)‐1*H*‐tetrazole (**16**), a known carboxylic acid isostere of proline, which resulted in a notable 90 % conversion. However, purification via column chromatography afforded only a 28 % isolated yield. Further experiments with increased amounts of aldehyde and ketone (up to four times the standard quantities) led to reduced conversion rates and a higher formation of undesired side products, such as the aldol product from the reaction of **9** and **10**.

Consequently, L‐proline (50 mol %) was the catalyst of choice for further optimization. Despite screening several solvents, e. g. MeOH, DMAc, *i*‐PrOH, ACN and 1,4‐dioxane, and various substrate equivalents under a range of temperatures (see supplementary information), we failed to identify conditions that offered the product in reasonable yields and purities. The best conditions identified in this first round of optimization were α‐hydroxyacetone (1.2 eq.) and formaldehyde (1.6 eq.) in DMSO as the solvent for 24 h at 45 °C; still, we only got approx. 35 % conversion of the starting material **8** of which only 50 % corresponded to the desired product **11**.

The main disadvantage of Mannich reactions, in general, is the overall reaction times, which are relatively long (up to 48 hours) under conventional methods.[^45^, ^49^] However, the use of microwave irradiation has been reported to speed up the conversion, thus maintaining or improving the yields.[^45^, ^46^, ^49^, ^50^, ^51^] Rodriguez and Bolm^[45]^ reported microwave‐assisted three‐component Mannich reactions in good to excellent yields (44–96 %) and in shorter reaction times (1–4 hours).^[45]^ We decided to explore the outcome of microwave irradiation on this proline‐catalyzed three‐component Mannich reaction, hoping to drive the reaction to completion in a shorter time (Table 1).

**Table 1:** Microwave‐assisted Mannich reaction.^[a]^

Entry	Solvent	mol % of catalyst	Temp (°C)	Time (hrs)	Conversion of SM (%)^[b]^
1	DMSO	50	45	1	35
2	DMAc	50	45	1	50^[c]^
3	DMSO	50	60	1	40^[c]^
4	DMSO	50	100	1	40^[c]^
5	DMSO	100	45	1	45^[c]^
6	DMSO	10	45	1	15
7	DMSO	50	45	0.5	10
8	DMSO	50	45	2	35
9	DMSO	50	45	1	38^[d]^
10	DMSO	50	45	1	40^[e]^
11	DMSO	50	45	1(4)^f^	98^[f,g]^

SM=starting material. Experimental conditions: under MW irradiation (power; 60 W and pressure 250 PSI) [a] mixture of 3‐fluoro‐4‐morpholinoaniline (100 mg, 0.5 mmol), aqueous formaldehyde (1.6 eq.), α‐hydroxyacetone (1.2 eq.) and L‐proline was allowed to react for 1 hour, reaction was monitored by LC–MS. [b] Desired product which was racemic. [c] Reaction favouring the formation of side products. [d] Only the catalyst was refreshed after 1 hour. [e] Catalyst and aldehyde were refreshed after 1 hour. [f] Catalyst, aldehyde and ketone were refreshed after every 1 hour for 4 hours. [g] Column chromatography was used for purification.

Promising results were obtained using DMSO with 50 mol % L‐proline at 45 °C (Table 1, entry 1) with the reaction favouring the formation of the desired product with a conversion of approx. 35 % after 1 hour. Results obtained for DMAc at 45 °C favoured the formation of impurities (Table 1, entry 2). Higher temperatures (60 and 100 °C) enhanced reaction conversions but also favoured the formation of side products (Table 1, entries 3 and 4). The effect of catalyst loadings using microwave irradiation was also evaluated (Table 1). The optimal results, favouring the formation of Mannich products, were obtained with 50 mol % of proline (Table 1, entry 1) which resulted in a conversion of 35 %. Increasing the amount of **9** gave better conversions (Table 1, entry 5), however, it favoured the formation of side products. Decreasing catalyst loading to 10 mol % provided lower reaction transformations, with less of the desired product (Table 1, entry 6).

Evidently, the reaction was not progressing due to one or more of the reagents being exhausted through side‐product formation. The reactions were monitored via LC–MS at 30, 60, and, 120 min intervals under the optimized conditions (Table 1, entries 7, 1 and 8 respectively). The transformation at 30 minutes was about 10 %, at 60 minutes 35 % and at 120 minutes remained at 35 %. Comparing these time points we then established that the reagents were exhausted in about 60 minutes. Refreshing the catalyst after the optimized time (Table 1, entry 9), showed only slight improvement in conversions from 35 to 38 %. However, when we continued to refresh the catalyst, aldehyde, and ketone after every hour for a total reaction time of four hours, we obtained 98 % conversion, the crude product mixture was separated by use of column chromatography to afford 50 % isolated yield of the desired 4‐(3‐fluoro‐4‐morpholinophenylamino)‐3‐hydroxybutan‐2‐one (**11**) as a racemate. Although proline no longer acted as a catalyst as we initially envisaged it should be noted that the reaction did not proceed in the absence of proline hence it is required to speed up the formation of the imine intermediate. Proline is an advantageous reagent considering it is a cheap, readily available, and ‘greener’ chemical compared to other routes to make these compounds. We also attempted the experiment starting with higher amounts of the aldehyde and ketone (2 and 4 times more). However, it resulted in lower conversion with more side products therefore we returned to refreshing the reagents as the optimum conditions.

Despite the desired mass of compound **11** observed on LC–M**S**, we noted two extra protons upon inspection of its proton NMR (see Supporting Information). This was attributed to the imine form of **11** due to the excess aldehyde in the reaction mixture. GC‐MS analysis further identified the imine mass. Thus, the subsequent addition of acid to the reaction mixture was required to hydrolyze the imine to the desired product **11** before column chromatography. It must be noted that the desired mass was observed during the reaction monitoring using LC–MS since the mobile phase is acidic. Furthermore, upon subjecting **11** to chiral HPLC, it proved to be racemic (see Supporting Information).

The only previously reported Mannich reaction involving formaldehyde, α‐hydroxyacetone, and an aniline (4‐methoxyaniline) yielded a 70 % enantiomeric excess (ee).^[46]^ Typically, 4‐methoxyaniline is preferred over aniline in organocatalyzed Mannich reactions due to its ability to significantly influence the enantio‐ and diastereoselectivity of the desired products.^[48]^ This suggests that the use of the non‐standard aniline, in this case, likely contributed to the observed lack of enantioselectivity. The proposed mechanism of this reaction involves proline forming an enamine with the ketone, which then attacks the imine generated from the reaction between aniline and the aldehyde. The chiral proline provides a stereocontrolled environment in this process. However, we postulate that, in our case, the use of a non‐standard aniline and microwave irradiation alters the reaction pathway. Proline may form an imine with either the aldehyde or the ketone, while the aniline could similarly react to form an imine with either the aldehyde or the enamine‐ketone intermediate. This multiplicity of pathways prevents the establishment of a selective chiral environment, leading to the observed lack of selectivity.

**Scheme 2:**
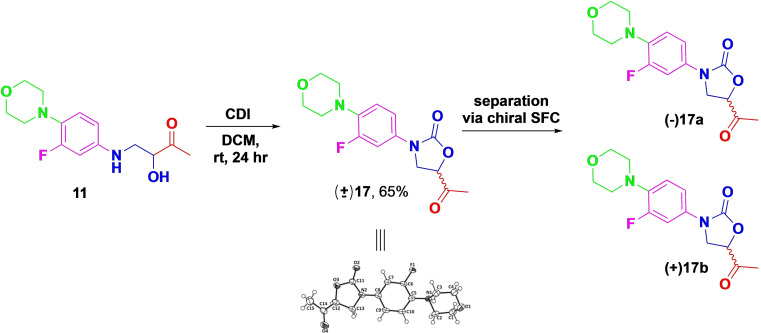
CDI cyclization and NaBH_4_ reduction reactions.

The Mannich product **11** was then effectively cyclized with CDI to create the oxazolidinone scaffold in DCM, yielding the desired novel racemic Linezolid derivative **17** (Scheme 2) with a favourable yield of 65 %. The racemic structure was further confirmed through X‐ray crystallography.^[52]^

**Scheme 3:**
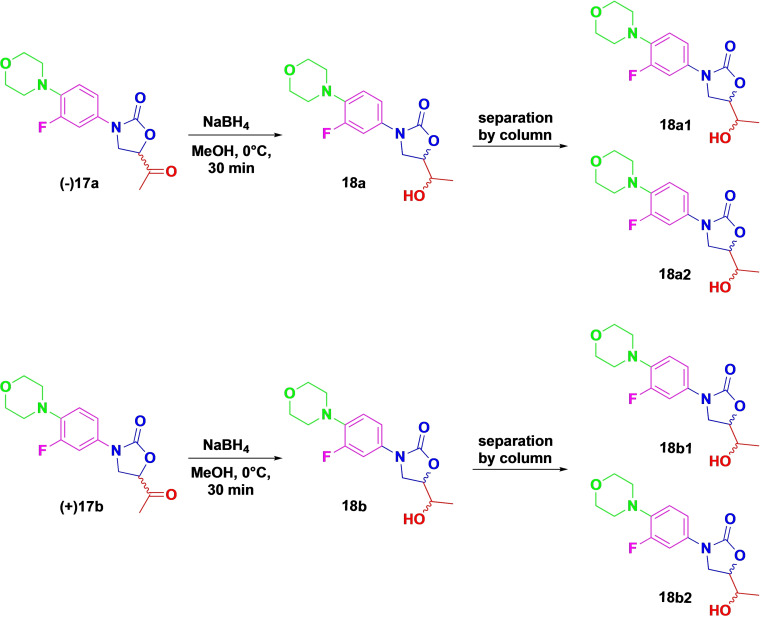
Reduction reaction of **17** and isolation of the diastereomers **18**.

Separating a racemic drug into its enantiomers is important for understanding and optimizing its biological activity.^[53]^ Thus, we separated the enantiomers of **17** using SFC equipped with a CHIRALPAK IA column, resulting in **17 a** and **17 b**. The respective enantiomers were further subjected to reduction using NaBH_4_ to form further analogues **18 a** and **18 b** (Scheme 3). Since this reduction created a second chiral centre, it resulted in two diastereomers per enantiomer, i. e. **18 a1–2** and **18 b1–2** (Scheme 3), separated using benchtop silica gel chromatography.

### 2.1 Biological Evaluation

In this study, we evaluated the newly synthesized oxazolidinone derivatives **17** and **18** for their potential to inhibit the growth of *M. smegmatis* PJV 53. The hindered drug development progress against *Mycobacterium tuberculosis* (*M. tb*) is attributed to its virulent nature and slow *in vitro* growth rate.^[54]^ Seeking a more expeditious approach to drug discovery for combating *M. tb*, researchers are turning to *M. smegmatis* as a surrogate organism in tuberculosis studies. This choice is motivated by *M. smegmatis*’ non‐pathogenic nature, faster growth rate, genetic similarity, and suitability for drug sensitivity testing.[^55^, ^56^] The enhanced growth rate facilitates swift experimentation and hypothesis testing in a laboratory environment under biosafety level 1 (BSL‐1) conditions. These observations have prompted the evaluation of the synthesized compounds on *M. smegmatis*, as an initial and rapid screening method for identifying potential anti‐TB agents.

We conducted antimicrobial susceptibility testing to determine the minimum inhibitory concentrations (MICs) of oxazolidinone derivatives **17** and **18**, utilizing the Clinical and Laboratory Standard Institute (CLSI) guidelines. As a reference, Rifampicin was employed as a control. Duplicate assays were performed to confirm the results, and the resulting MICs are presented in Table 2.

**Table 2:** MICs of synthesized oxazolidinones against *M. smegmatis*.

Compounds	*M. smegmatis* PJV 53
	MIC (mg/L)
**17**	32
**17 a**	16
**17 b**	No activity
**18 a1**	8
**18 a2**	32
**18 b1**	No activity
**18 b2**	No activity
Rifampicin	8

To our delight the racemic compound **17**, which was the initial oxazolidine derivative provided an excellent starting point, as a MIC of 32 mg/L against *M. smegmatis* was observed (Table 2). Upon separating this racemate into its enantiomers **17 a** and **17 b**, the activity was 16 mg/L and no activity, respectively.

Diastereomer (**18 a1**) exhibited excellent potency, producing an 8 mg/L MIC correlating to the reference drug, Rifampicin. The observed disparity in MIC values between diastereomers **18 a1** and **18 a2** highlights the complex relationship between chemical structure chirality and its effectiveness against *M. smegmatis*. In contrast, neither **18 b1** nor **18 b2** showed any inhibitory activity against *M. smegmatis* **PJV53**. This suggests that the stereochemical arrangement of compound **18 b** may not be suitable for antibacterial activity.

Importantly, the reduction of ketone **17** to alcohol **18** resulted in a 4‐fold decrease in inhibitory activity. This suggests the significance of the ketone moiety, for enhanced activity and hints that the alcohol group may be overly polar, hindering effective permeation through the lipid‐rich bacterial cell wall. Both compounds **17** and **18** present promising targets for future functionalization or conjugation studies.

## 3 Conclusions

This work demonstrates a streamlined, three‐step process for synthesizing oxazolidinone analogues using readily available materials. The L‐proline‐mediated three‐component Mannich reaction, especially under microwave irradiation, proved significantly more efficient than conventional methods. The synthesized oxazolidinone compounds showed moderate to strong antibacterial activity against *M. smegmatis*, with MIC values between 8 and 32 mg/L. Notably, compound **18 a1** exhibited exceptional inhibitory activity and was comparable with the commercial Rifampicin, thus making it a promising candidate for further optimization in antimicrobial drug development. The observed decrease in MIC values by resolving the enantiomers and corresponding diastereomers highlighted the complex relationship between chemical structure chirality and effectiveness against microorganisms. Despite using stoichiometric amounts of proline without enantioselectivity, this method offers a simplified and time‐efficient route to highly valuable oxazolidinone derivatives. It also lays the foundation for future research to enhance specificity and introduce enantioselective improvements.

## Experimental

### Materials and Reagents

Reagents and solvents were either purchased from Aldrich or Merck suppliers. For thin‐layer chromatography (TLC), silica gel plates Merck 60 F254 were used, and compounds were visualized by irradiation with UV light. Flash chromatography was performed using silica gel Merck 60 (particle size 0.040–0.063 mm). ^1^H NMR and ^13^C NMR spectra were recorded on Bruker Advance III 400 MHz instrument at room temperature in CDCl_3_ using the residual solvent signal as a reference. ^13^C NMR is the APT experiment. The coupling constants (J) are given in Hz. LC–MS was performed on all synthesized compounds using a Shimadzu LCMS2020 system with a C_18_ column and acetonitrile and water as the mobile phase. GC–MS analysis was performed on a Shimadzu GC‐2010 equipped with a GCMS‐QP2010 mass spectrometer and a Zebron ZB‐1MS column with Helium as the carrier gas. Separation of enantiomers was done on a Sepiatec Prep SFC basic instrument. HRMS data were obtained using a Bruker micrOTOF‐Q II instrument operating at ambient temperature and a sample concentration of approximately 1.0 ppm. Microwave‐assisted reactions were carried out on a CEM Discover SP system. The enantiomeric/diastereomeric excess of the products was determined on an Agilent 1100 HPLC equipped with a YMC CHIRALART Cellulose‐SC or CHIRALPAK IA column. The bacterial strain used in the MIC experiments, *Mycobacterium smegmatis* PJV 53, was obtained from the Microbiology Laboratory at the University of KwaZulu‐Natal.

### Chemistry

#### General Procedure for Synthesis of 4‐(3‐Fluoro‐4‐Morpholinophenylamino)‐3‐Hydroxybutan‐2‐One (11)

Under MW irradiation (temp=45 °C, power=60 W and pressure=250 PSI) a mixture of 3‐fluoro‐4‐morpholinoaniline (100 mg, 0.5 mmol), formaldehyde (1.6 eq, 36 % aqueous solution), α‐hydroxyacetone (1.2 eq.) and L‐proline (50 mol %) in DMSO (2 mL) was stirred for one hour. Aldehyde, ketone, and catalyst were refreshed after the hour was complete, and the reaction was placed under MW irradiation again and stirred for one hour. This process was repeated, such that the reagents were refreshed three times, and the total time under MW irradiation was 4 hours. Upon completion of the reaction, as determined by LC–MS, the reaction mixture was diluted with water (10 mL) and then extracted with DCM (3×15 mL). The combined organic layers were dried with anhydrous MgSO_4,_ and the solvent was removed under reduced pressure. To this residue was added a solution consisting of 5 mL of 2 M HCl and 10 mL of methanol. This mixture was allowed to stir overnight. Upon completion as determined by GC–MS, the acid was neutralized using saturated NaHCO_3_. The resulting aqueous solution was then extracted with DCM (3×15 mL), and the combined organic layers were dried with anhydrous MgSO_4_ and reduced under pressure. The crude mixture was then purified by column chromatography to afford **11** as a yellow‐brown oil. The *ee* of **11** was determined by HPLC analysis using a YMC CHIRALPAK Cellulose‐SC column, measured at 254 nm, with a mobile phase of 90 : 10 hex/i‐PrOH at 1 mL/min. TLC (EtOAc/hexane: 40 : 60, R_f_=0.4); Yield 50 %; ^1^H NMR (400 MHz, CDCl_3_): δ 7.43 (dd, *J*_*H*−*H*_=8.22, 2.5 Hz, 1H), 7.11 (dd, *J*=8.65, 2.4 Hz, 1H), 6.95 (t, *J*=9.26 Hz, 1H), 4.87 (t, *J*=7.7 Hz 1H), 4.15 (d, *J*=8.96 Hz, 2H), 3.88 (t, *J*=9.03 Hz, 4H), 3.07 (t, *J*=3.95 Hz, 4H), 2.43 (s, 3H). ^13^C NMR (400 MHz, CDCl_3_): δ 204.9 (CO), 153.4 (CF), 132.6 (CH), 118.9 (CH), 114.5 (CH), 108.1 (CH), 75.3 (CO), 66.9 (CH_2_), 51.5 (CH_2_), 46.8 (CH_2_), 26.1 (CH_3_). HRMS (ESI^+^) *m/z* calcd. for C_14_H_19_FN_2_O_3_ [M+H]^+^: 283.1456; found 283.1469

#### General Procedure for Synthesis of Racemic 5‐Acetyl‐3‐(3‐Fluoro‐4 Morpholinophenyl)oxazolidin‐2‐One (17)

A solution of the **11** (100 mg, 0.4 mmol) and CDI (1.2 eq.) in DCM (10 mL) was stirred at room temperature for 24 hours. Upon completion of the reaction as determined by LC–MS, the solvent was evaporated under reduced pressure and the resulting residue was purified via column chromatography to afford **17**. The *ee* of **17** was determined by HPLC analysis using a CHIRALART IA column, measured at 254 nm, with a mobile phase of 90 : 10 hex/i‐PrOH at 1 mL/min. Enantiomers of **17** were separated by SFC equipped with a CHIRALART IA column and using 10 % MeOH as a modifier at a flow rate of 10 ml/min (**17 a** Rt=21.1 min, **17 b** Rt=24.6 min). Both enantiomers were isolated as white solids; TLC (EtOAc/hexane: 30 : 70, R_f_=0.5); Combined Yield 65 %; **17 a**: [α]^20^_D_ −5.82 (MeOH, *c*=0.9). **17 b**: [α]^20^_D_+5.82 (MeOH, *c*=0.9). ^1^H NMR (400 MHz, CDCl_3_): δ 7.43 (dd, *J*=9.5, 1.56 Hz, 1H), 7.10 (dd, *J*=6.1, 1.4 Hz, 1H), 6.93 (t, *J*=6.08 Hz, 1H), 4.84 (t, *J*=5.28 Hz, 1H), 4.12 (d, *J*=5.2 Hz, 2H), 3.85 (t, *J*=3.08 Hz, 4H), 3.05 (t, *J*=3.04 Hz, 4H), 2.41 (s, 3H). ^13^C NMR (400 MHz, CDCl_3_): δ 204.7 (CO), 156.7 (CF), 155.0 (CO), 153.4 (CH), 133.1 (CH), 132.5 (CH), 119.6 (CH), 113.9 (CH), 108.4 (CH), 75.2 (CH), 68.0 (CH_2_), 51.3 (CH_2_), 47.4 (CH_2_), 27.4 (CH_3_); HRMS (ESI^+^) *m/z* calcd. for C_15_H_18_FN_2_O_4_ [M+H]^+^: 309.1245; found 309.1260

#### General Procedure for Synthesis of Diastereomers 3‐(3‐Fluoro‐4‐Morpholinophenyl)‐5‐(1‐Hydroxyethyl)Oxazolidin‐2‐One (18 a and 18 b)

To the reaction mixture of **17** (400 mg, 1.3 mmol) in MeOH (10 mL), 3 eq. of NaBH_4_ was added in one portion at 0 °C. The reaction was stirred for 30 minutes, after which brine (15 mL) was added, and the mixture was extracted with EtOAc (3×15 mL). The combined organic layers were dried over anhydrous MgSO_4_, evaporated under reduced pressure and then purified by column chromatography to give the corresponding reduced alcohols **18 a** and **18 b** (yield 90 % overall). The *d.r* of **18 a** and **18 b** were determined by HPLC using a YMC CHIRALART Cellulose‐SC column, measured at 254 nm, with a mobile phase of hex/i‐PrOH 80 : 20 at 1 mL/min.

#### 3‐(3‐Fluoro‐4‐Morpholinophenyl)‐5‐(1‐Hydroxyethyl)Oxazolidin‐2‐One (18 a)

White solid, TLC (EtOAc/hexane: 30 : 70, R_f_=0.4), 45 % yield. **18a1**: [α]^20^_D_‐73.8 (DMSO/H_2_O, *c*=0.228) and **18a2**: [α]^20^_D_ −89.1 (DMSO/H_2_O, *c*=0.17). ^1^H NMR (400 MHz, CDCl_3_): δ 7.39 (dd, *J*=14.40, 2.63 Hz, 1H), 7.13 (dd, *J*=9.92, 1.56 Hz, 1H), 6.92 (t, *J*=9.16 Hz, 1H), 4.50–4.45 (m, 1H), 4.14–4.11 (m, 1H), 4.00 (t, *J*=7.60 Hz, 1H), 3.86 (t, *J*=4.65 Hz, 4H), 3.04 (t, *J*=4.64 Hz, 4H), 1.24 (d, *J*=6.48 Hz 3H). ^13^C NMR (400 MHz, CDCl_3_): δ 156.7 (CF), 154.7 (CO), 154.3 (CH), 136.3 (CH), 133.3 (CH), 118.8 (CH), 113.9 (CH), 107.6 (CH), 75.9 (CH), 68.2 (CO), 67.0 (CH_2_), 66.7 (CH_2_), 51.0 (CH_2_), 45.3 (CH_2_), 17.7 (CH_3_). HRMS (ESI^+^) *m/z* calcd. for C_15_H_20_FN_2_O_4_ [M+H]^+^: 311.1402; found 311.1403

#### 3‐(3‐Fluoro‐4‐Morpholinophenyl)‐5‐(1‐Hydroxyethyl)Oxazolidin‐2‐One (18 b)

White solid, TLC (EtOAc/hexane: 30 : 70, R_f_=0.3), 45 % yield. **18b1**: [α]^20^_D_ −53.7 (DMSO/H_2_O, *c*=0.17). **18b2**: [α]^20^_D_ −36.2 (DMSO/H_2_O, *c*=0.176). ^1^H NMR (400 MHz, CDCl_3_): δ 7.36 (dd, *J*=14.36, 2.56 Hz, 1H), 7.06 (dd, *J*=8.72, 2.12 Hz, 1H), 6.84 (t, *J*=9.17 Hz, 1H), 4.43–4.41 (m, 1H), 4.40–4.38 (m, 2H), 3.79 (t, *J*=4.64 Hz, 4H), 2.97 (t, *J*=4.68 Hz, 4H), 1.18 (d, *J*=6.52 Hz, 3H). ^13^C NMR (CDCl_3_): δ 156.8 (CF), 154.7 (CO), 154.3 (CH), 135.8 (CH), 133.6 (CH), 119.3 (CH), 114.1 (CH), 107.5 (CH), 75.0 (CH), 68.2 (CH_2_), 66.9 (CO), 51.0 (CH_2_), 45.5 (CH_2_), 18.1 (CH_3_). HRMS (ESI^+^) *m/z* calcd. for C_15_H_20_FN_2_O_4_ [M+H]^+^: 311.1402; found 311.1390

### MIC Assay for M. Smegmatis

#### Compounds and Reagents

Compounds **17 a–b** and **18 a–b** were dissolved in 50 % DMSO (final DMSO concentration was <10 %). The mycobacterium strain used was first cultured on Middlebrook 7H11 agar (Difco^TM^), supplemented with 10 % (v/v) Oleic acid Albumin Dextrose Catalase (OADC) enrichment and glycerol for 48 to 72 hours at 37 °C prior the experiments were employed. This strain was cultured in Middlebrook 7H9 broth (Difco^TM^) supplemented with 10 % (v/v), OADC, 0.05 % (wt/v) Tween 80 and glycerol according to the manufacturer's instructions. Rifampicin was used as the reference drug.

#### Minimum Inhibitory Concentration (MIC) Determination

The MIC of Rifampicin and the oxazolidinone derivatives were determined using the broth microdilution method according to the CLSI guidelines.^[57]^ Briefly, two‐fold dilutions of each drug/compound were made in Middlebrook 7H9 broth in a 96‐well microtitre plate. A 0.5 McFarland standardized bacterial suspension equivalent to 1×10^6^ cfu/mL was added in the volume of 20 μL, to obtain a final volume of 200 μ l in each microtitre well. The plates were then incubated for 72 hours at 37 ± 2 °C under aerobic conditions. Resazurin dye (20 μL) was added to each microtiter well to visualize the MIC. The MIC was then recorded as the lowest concentration, at which there was no visible growth. Control wells included the amount of DMSO used for dissolving the compound. Both positive and negative controls of *M. smegmatis* and 7H9 broth were made in each plate. The assay was performed in triplicate to confirm the results.

## Conflict of Interests

The authors declare no conflict of interest.

## Supporting information

As a service to our authors and readers, this journal provides supporting information supplied by the authors. Such materials are peer reviewed and may be re‐organized for online delivery, but are not copy‐edited or typeset. Technical support issues arising from supporting information (other than missing files) should be addressed to the authors.

Supporting Information

## Data Availability

The data that support the findings of this study are available from the corresponding author upon reasonable request.
