# Supplementary material for: Facile Synthesis of Oxazolidinones as Potential Antibacterial Agents
Source: ChemistryOpen. 2025 Jan 7;14(7):e202400432. doi: 10.1002/open.202400432 (PMC12256933; doi:10.1002/open.202400432)

ChemistryOpen

Supporting Information

Facile Synthesis of Oxazolidinones as Potential Antibacterial Agents

Secret P. Els, Kimberleigh B. Govender, Mxolisi K. Sokhela, Nilay Bhatt, Nakita Reddy, Hendrik G. Kruger, Per I. Arvidsson, Hendra Gunosewoyo, Thavendran Govender, and Tricia Naicker*

SUPPLEMENTARY INFORMATION

Facile Synthesis of Oxazolidinones as Potential Anti-bacterial Agents

Secret P. Els,[a] Kimberleigh B. Govender,[a] Mxolisi K. Sokhela,[a] Nilay Bhatt,[a] Nakita Reddy,[a] Hendrik G. Kruger,[a] Per I. Arvidsson,[a, b] Hendra Gunosewoyo,[c] Thavendran Govender[d] and Tricia Naicker* [a]

[a] *Department of Pharmaceutical Chemistry Catalysis and Peptide Research Unit, University of KwaZulu-Natal, Durban 4000, South Africa*

[b] *Science for Life Laboratory, Drug Discovery & Development Platform & Division of Translational Medicine and Chemical Biology, Department of Medical Biochemistry and Biophysics, Karolinska Institute, Stockholm, Sweden*

[c] *School of Pharmacy and Biomedical Sciences, Faculty of Health Sciences, Curtin University*

[d] *Department of Chemistry, University of Zululand, Private Bag X1001, KwaDlangezwa 3886, South Africa*

Corresponding author. Tel.: +27 312608942/1845; mobile: +27 827729578

Email address: Naickert1@ukzn.ac.za (T Naicker).

1) Experimental

General procedure for the solvent screen of the three-component Mannich reaction under conventional method

A mixture of 3-fluoro-4-(4-morpholinyl)-aniline (100 mg, 0.5 mmol), formaldehyde (1.2 eq., 36% aqueous), α-hydroxyacetone (1.2 eq.) and L-proline (50 mol %) in 10 mL DMSO was stirred for 72 hours at ambient temperature. The reaction was monitored by LCMS. Results are shown below in Table 1.

Table S1. Solvent screen for L-proline catalysed three component Mannich reaction[a]

Entry	Solvent	Ketone (mol eq.)	Aldehyde (mol eq.)	Conversion (%)
1	DMSO	1.2	1.2	20[b]
2	MeOH	1.2	1.2	50[b]
3	DMAc	1.2	1.2	25
4	IPA	1.2	1.2	5[b]
5	ACN	1.2	1.2	5[b]
6	1.4 dioxane	1.2	1.2	5[b]
7	DMSO	2.5	1.2	35[b]
8	DMSO	0.5	1.2	10
9	DMSO	1.2	1.6	30
10	MeOH	1.2	1.6	56[b]
11	DMAc	1.2	1.6	30
12	DMSO	1.2	1.6	33[c]
13	DMAc	1.2	16	42[b, c]
14	DMSO	1.2	1.6	36[b, d]
15	DMSO	1.2	1.6	40[b, e]

[a]Experimental conditions: a mixture of 3-fluoro-4-morpholinoaniline (0.5 mmol, 1 eq), aq. formaldehyde (0.6 mmol), α-hydroxyacetone (0.6 mmol) and l-proline (50 mol %) was stirred at room temperature for 72 hours. Reaction progress was monitored by LC-MS. [b] Reaction favoured the formation of by-products. [c]Reaction was conducted at 45°C. [d]Reaction was conducted at 60°C. [e]Reaction was conducted at 100°C.

.

2) Spectral Data

Chiral HPLC spectra

S2. HPLC spectra of 4-(3-Fluoro-4-morpholinophenylamino)-3-hydroxybutan-2-one (11)

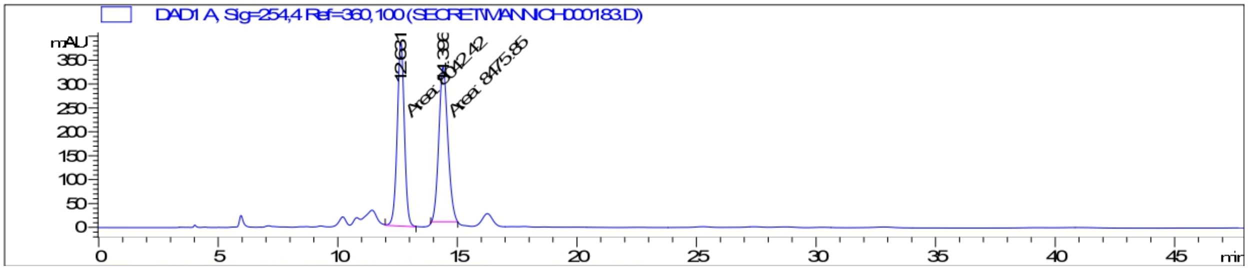

Signal 1: DAD1 A, Sig=254,4 Ref=360,100

Peak #	RetTime [min]	Type	Width [min]	Area [mAU*s]	Height [mAU]	Area %
1	12.631	MM	0.3480	8042.42187	385.19919	48.6880
2	14.396	MM	0.4366	8475.84863	323.53088	51.3120

Totals : 1.65183e4 708.73007

S3. HPLC spectra of racemic 5-acetyl-3-(3-fluoro-4 morpholinophenyl)oxazolidin-2-one (17)

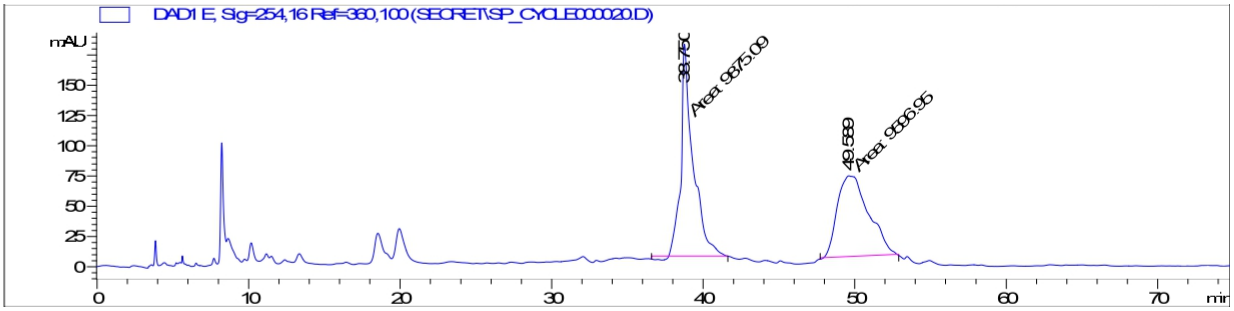

Signal 2: DAD1 E, Sig=254,16 Ref=360,100

Peak #	RetTime [min]	Type	Width [min]	Area [mAU*s]	Height [mAU]	Area %
1	38.750	MM	0.9388	9875.09277	175.30905	50.7142
2	49.589	MM	2.3931	9596.95020	66.83752	49.2858

Totals : 1.94720e4 242.14658

S4. HPLC spectra of (–)-5-acetyl-3-(3-fluoro-4 morpholinophenyl)oxazolidin-2-one after SFC purification (17a)

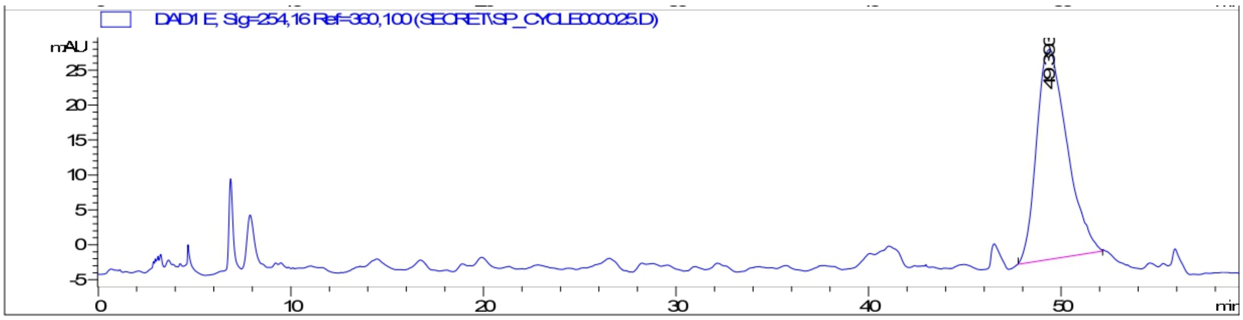

S5. HPLC spectra of (+)-5-acetyl-3-(3-fluoro-4 morpholinophenyl)oxazolidin-2-one after SFC purification (17b)

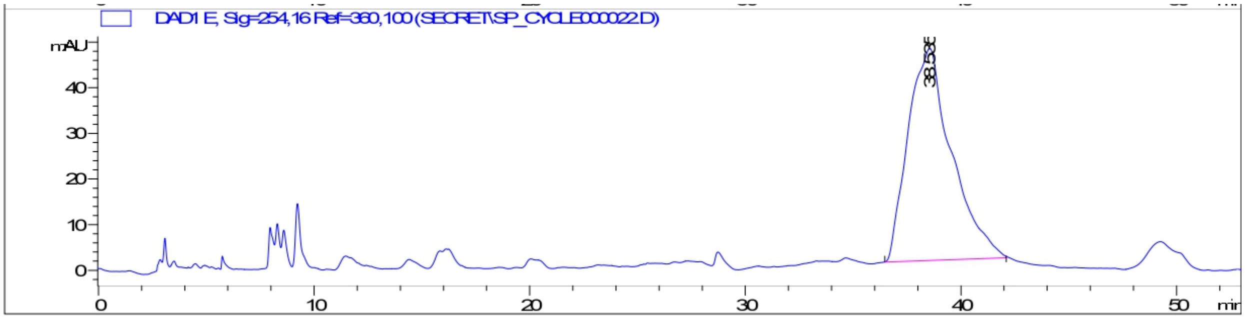

S6. HPLC spectra of 3-(3-fluoro-4-morpholinophenyl)-5-(1-hydroxyethyl)oxazolidin-2-one (18a1 and 18a2)

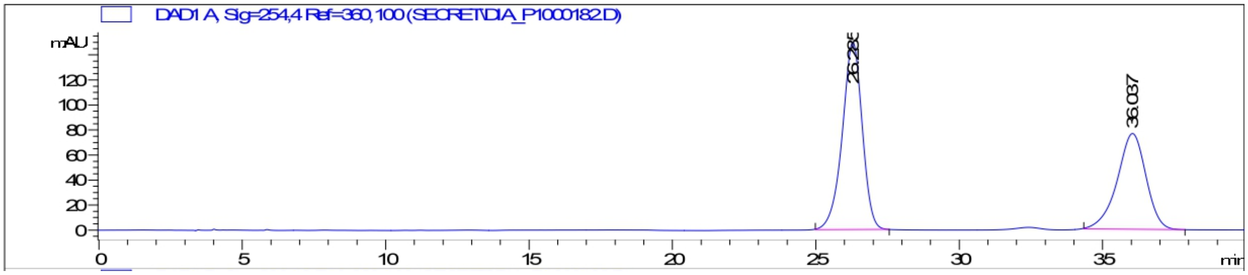

Signal 1: DAD1 A, Sig=254,4 Ref=360,100

Peak #	RetTime [min]	Type	Width [min]	Area [mAU*s]	Height [mAU]	Area %
1	26.285	BB	0.7359	7242.66016	149.85141	57.4752
2	36.037	BB	1.0464	5358.69775	76.58160	42.5248

Totals : 1.26014e4 226.43301

S7. HPLC spectra of 3-(3-fluoro-4-morpholinophenyl)-5-(1-hydroxyethyl)oxazolidin-2-one (18b1 and 18b2)

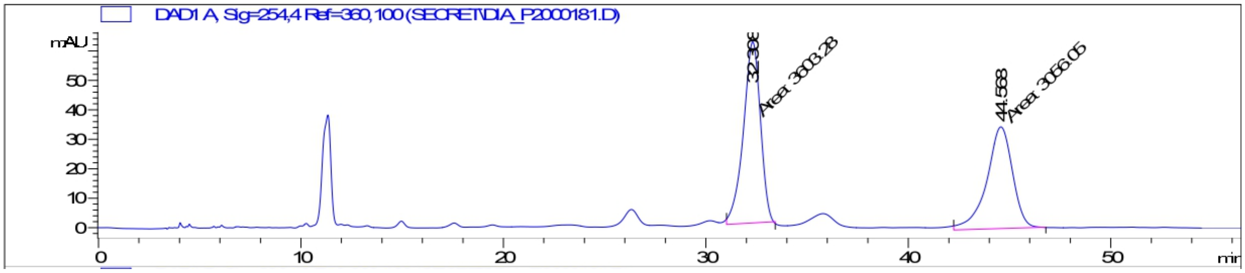

Signal 1: DAD1 A, Sig=254,4 Ref=360,100

Peak #	RetTime [min]	Type	Width [min]	Area [mAU*s]	Height [mAU]	Area %
1	32.308	MM	0.9767	3603.28320	61.48659	54.1088
2	44.568	MM	1.4758	3056.04858	34.51396	45.8912

Totals : 6659.33179 96.00055

NMR spectra

S8. Crude ^{1}H NMR of imine by-product

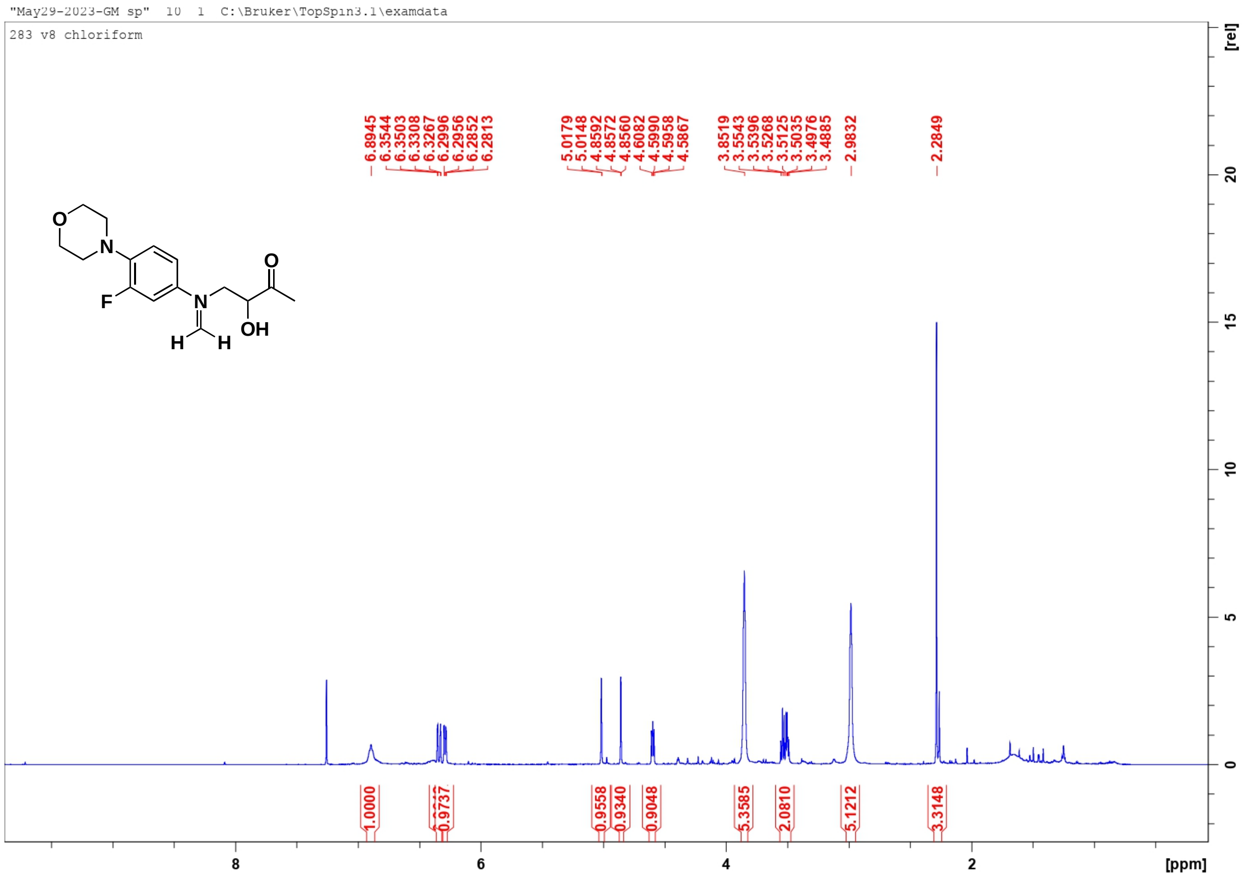

S9. ^{1}H NMR of 4-(3-Fluoro-4-morpholinophenylamino)-3-hydroxybutan-2-one (11)

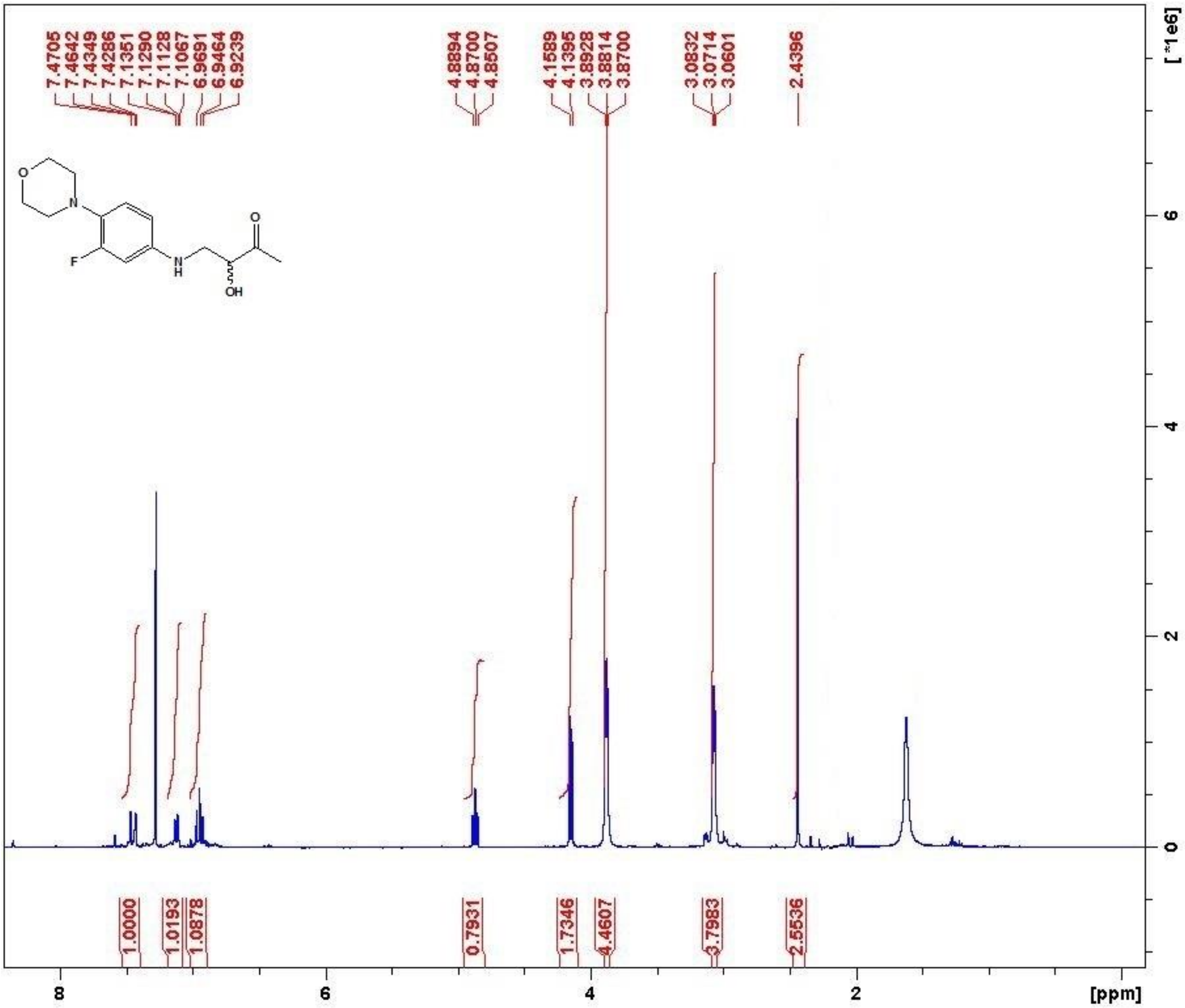

S10. ^{13}C APT NMR of 4-(3-Fluoro-4-morpholinophenylamino)-3-hydroxybutan-2-one (11)

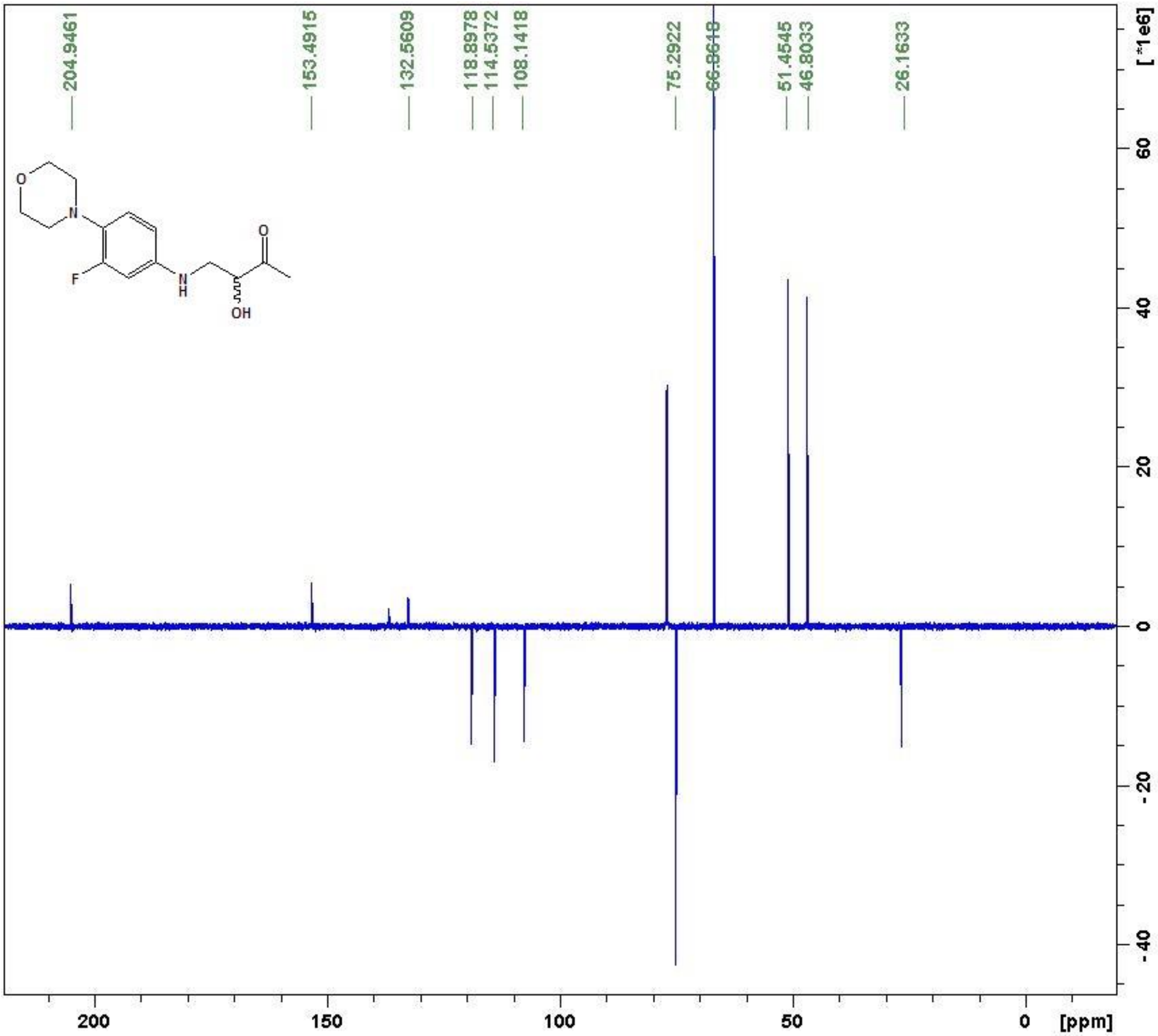

S11. ^{1}H NMR of Racemic 5-acetyl-3-(3-fluoro-4 morpholinophenyl)oxazolidin-2-one (17)

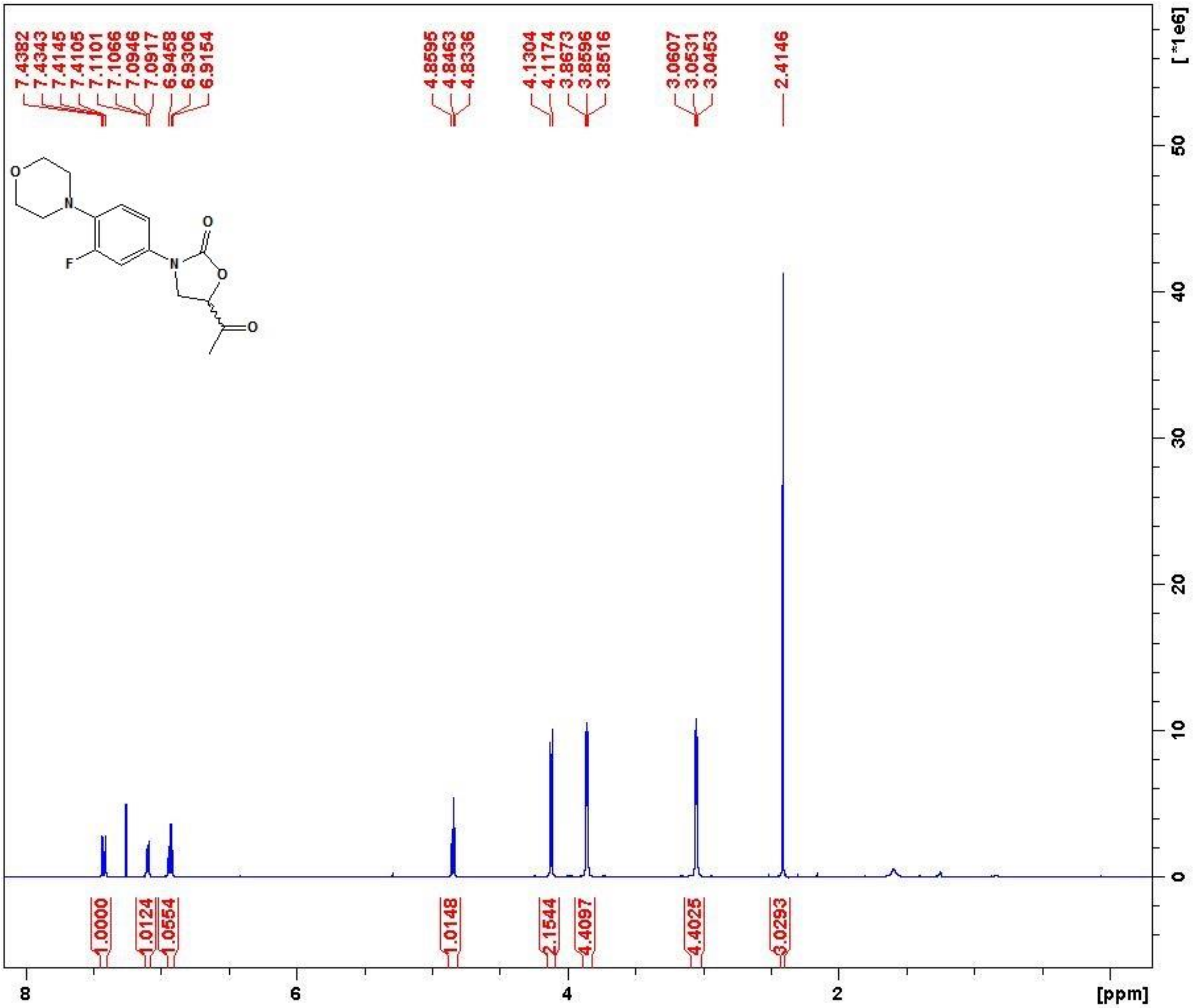

S12. ^{13}C APT NMR of Racemic 5-acetyl-3-(3-fluoro-4 morpholinophenyl)oxazolidin-2-one (17)

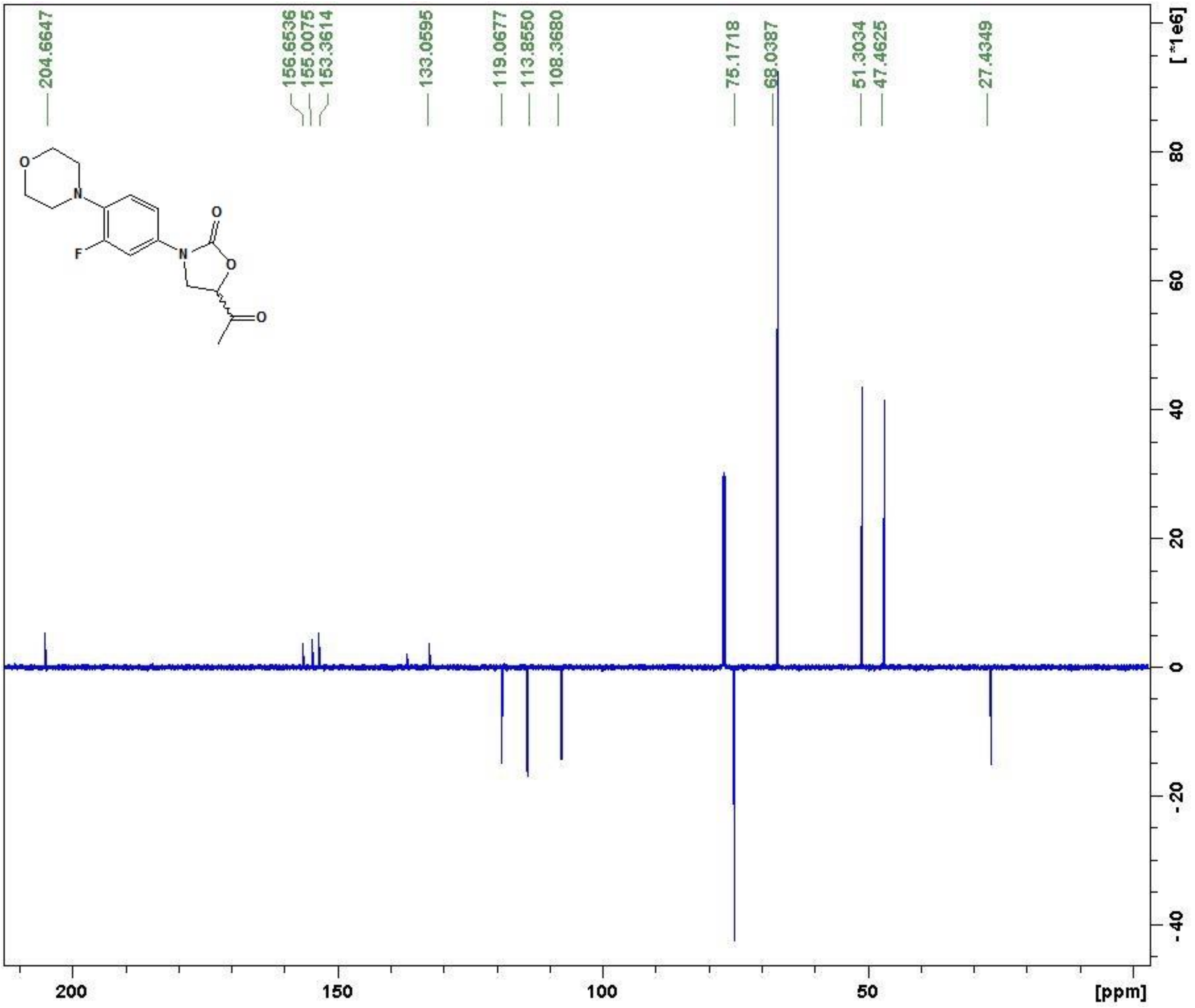

S13. ^{1}H NMR of 3-(3-fluoro-4-morpholinophenyl)-5-(1-hydroxyethyl)oxazolidin-2-one (18a)

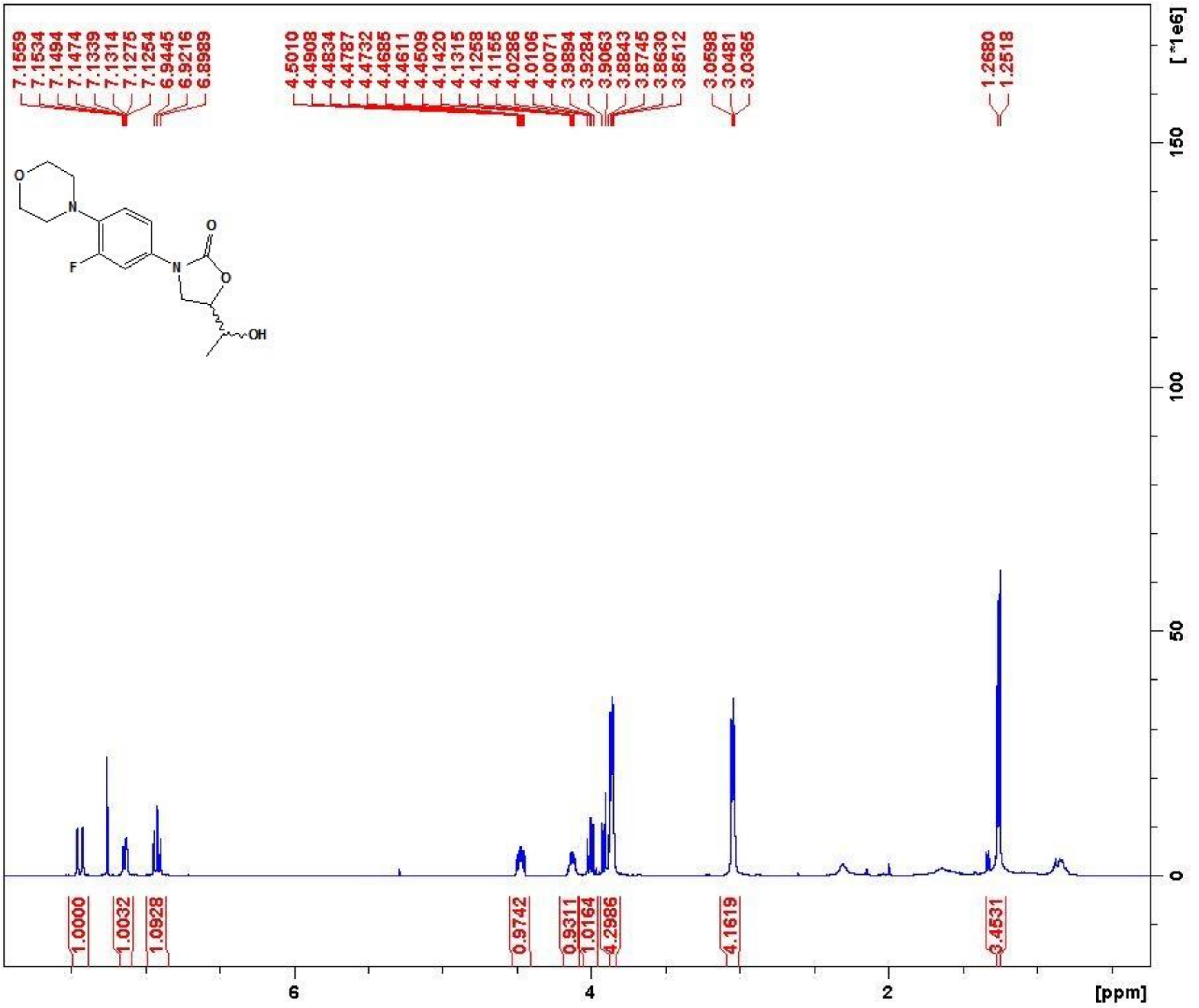

S14. ^{13}C APT NMR of 3-(3-fluoro-4-morpholinophenyl)-5-(1-hydroxyethyl)oxazolidin-2-one (18a)

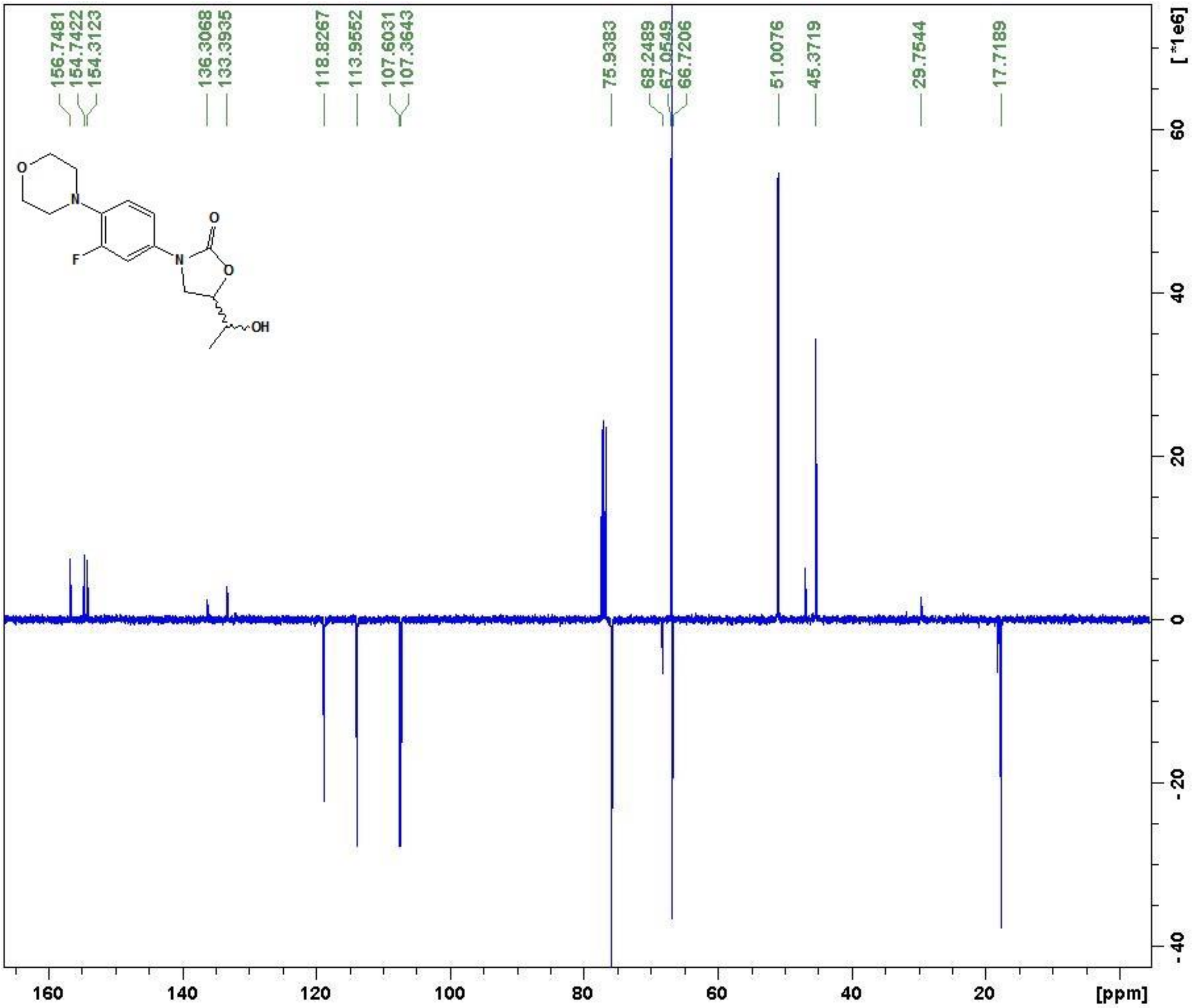

S15. ^{1}H NMR of 3-(3-fluoro-4-morpholinophenyl)-5-(1-hydroxyethyl)oxazolidin-2-one (18b)

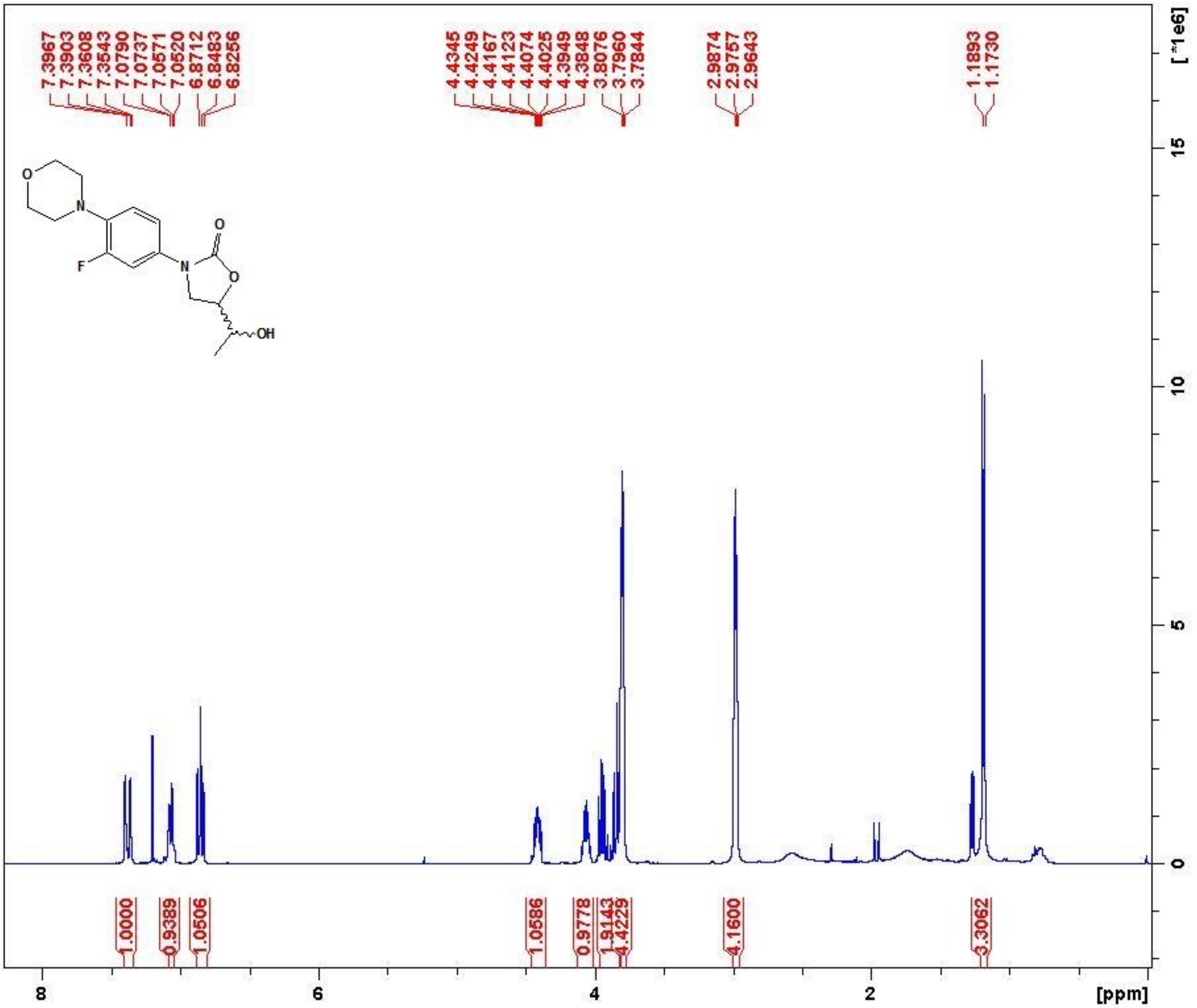

S16. ^{13}C APT NMR of 3-(3-fluoro-4-morpholinophenyl)-5-(1-hydroxyethyl)oxazolidin-2-one (18b)

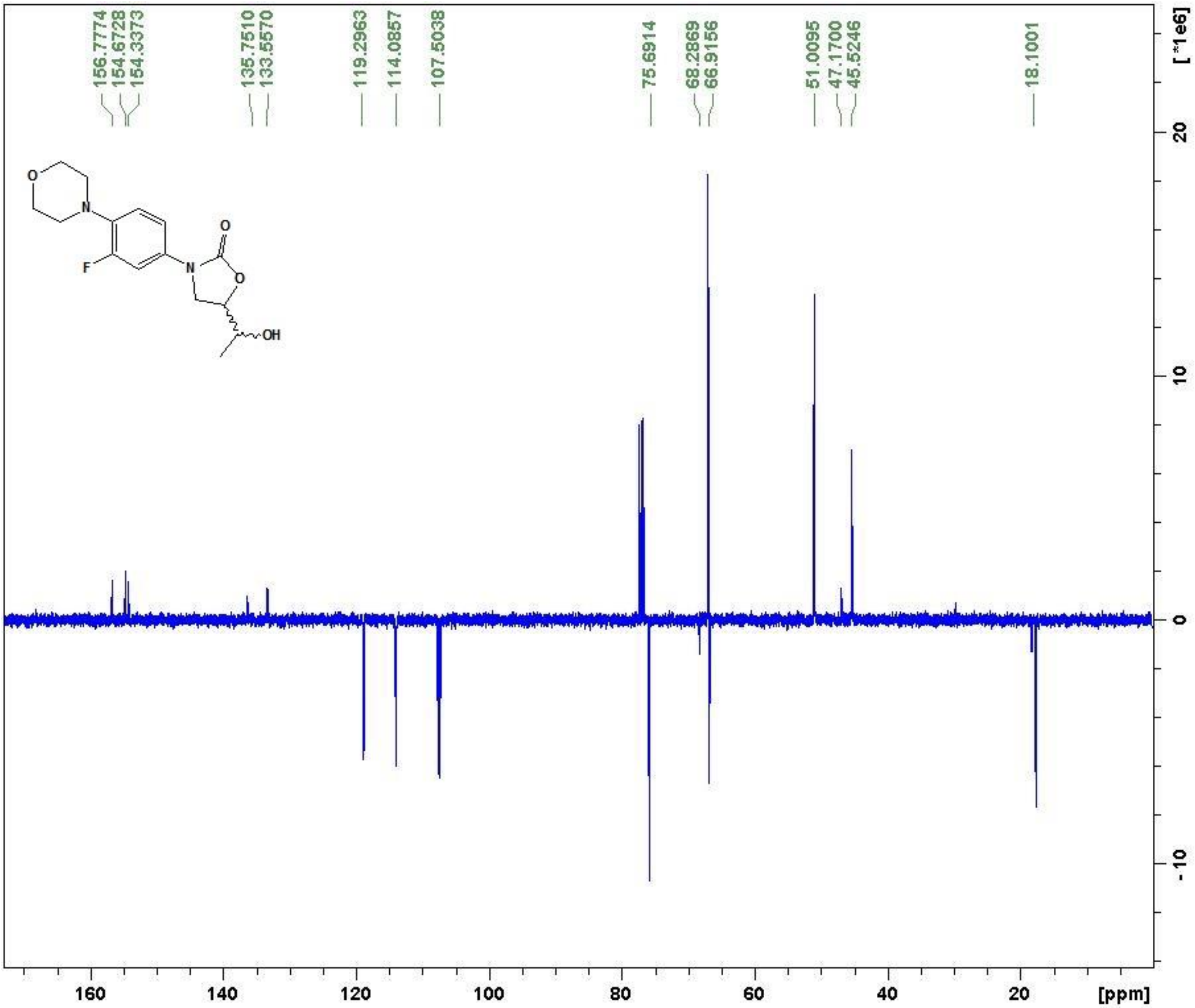

HRMS spectra of novel compounds

S17. HRMS of 4-(3-fluoro-4-morpholinophenylamino)-3-hydroxybutan-2-one (11)

Display Report

Analysis Info

Analysis Name	D:\Data\YAHYA\Sox_5_85_01_7314.d	Acquisition Date	10/1/2015 11:10:15 AM
Method	fia.m	Operator	BDAL@DE
Sample Name	Sox_5	Instrument	micrOTOF-Q 10139
Comment			

Acquisition Parameter

Source Type	ESI	Ion Polarity	Positive	Set Nebulizer	0.4 Bar
Focus	Not active	Set Capillary	4500 V	Set Dry Heater	200 °C
Scan Begin	100 m/z	Set End Plate Offset	-500 V	Set Dry Gas	4.0 l/min
Scan End	2300 m/z	Set Collision Cell RF	600.0 Vpp	Set Divert Valve	Source

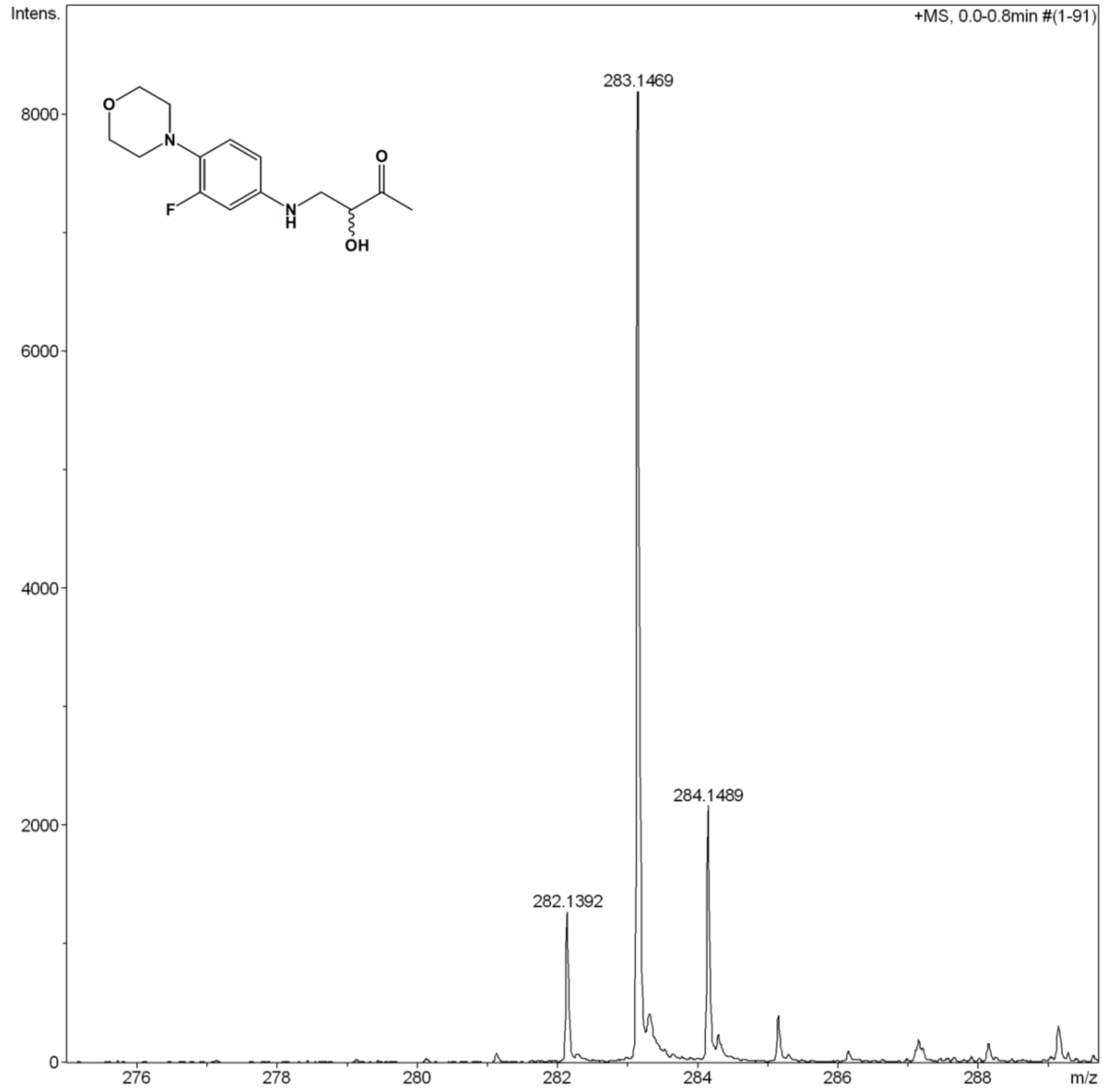

S18. HRMS of Racemic 5-acetyl-3-(3-fluoro-4 morpholinophenyl)oxazolidin-2-one (17)

Display Report

Analysis Info

Analysis Name	D:\Data\YAHYA\Sox_4_84_01_7312.d	Acquisition Date	10/1/2015 11:04:05 AM
Method	fia.m	Operator	BDAL@DE
Sample Name	Sox_4	Instrument	micrOTOF-Q 10139
Comment			

Acquisition Parameter

Source Type	ESI	Ion Polarity	Positive	Set Nebulizer	0.4 Bar
Focus	Not active	Set Capillary	4500 V	Set Dry Heater	200 °C
Scan Begin	100 m/z	Set End Plate Offset	-500 V	Set Dry Gas	4.0 l/min
Scan End	2300 m/z	Set Collision Cell RF	600.0 Vpp	Set Divert Valve	Source

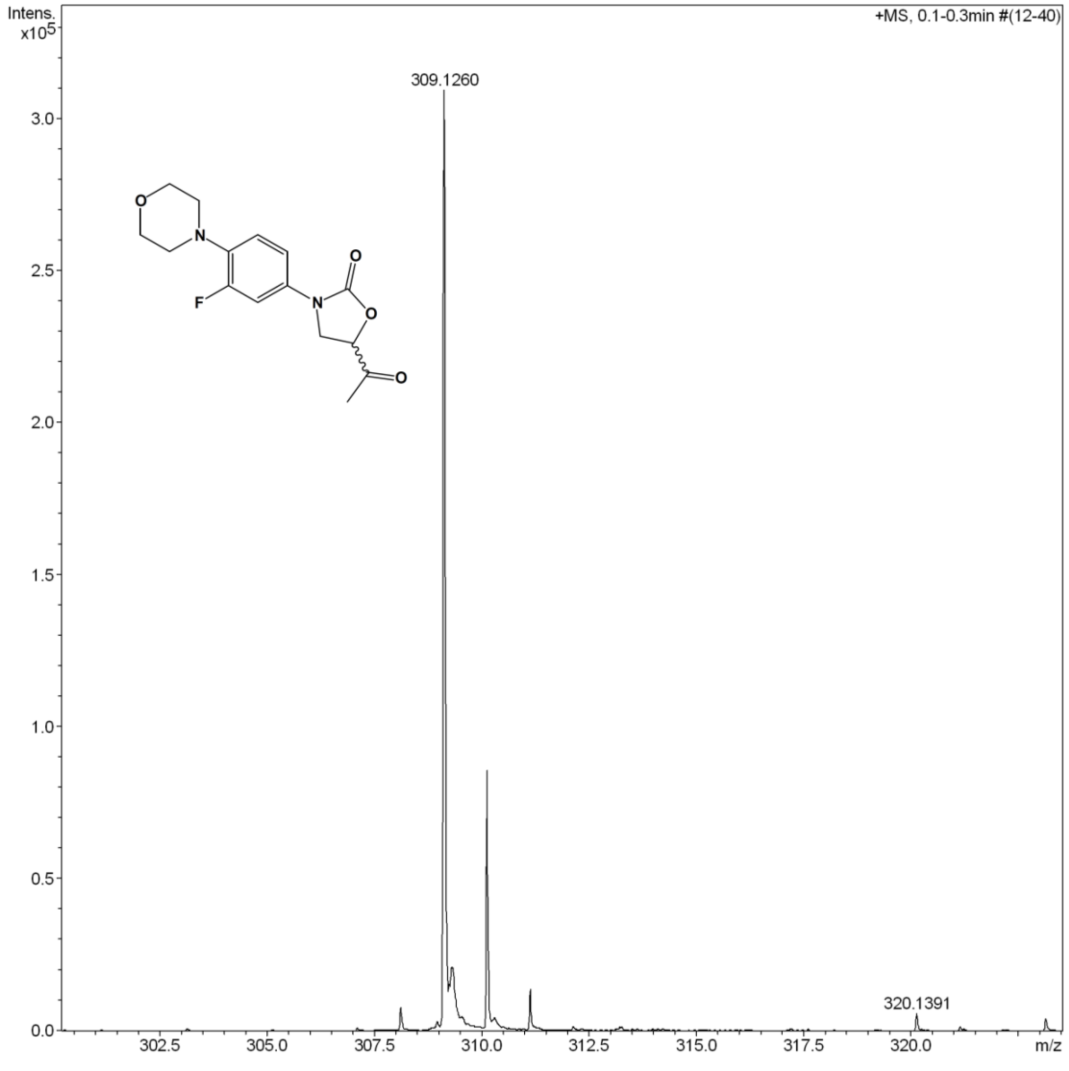

S19. HRMS of 3-(3-fluoro-4-morpholinophenyl)-5-(1-hydroxyethyl)oxazolidin-2-one (18a)

Display Report

Analysis Info

Analysis Name	D:\Data\YAHYA\Sox_2_82_01_7326.d	Acquisition Date	10/7/2015 11:31:02 AM
Method	fia.m	Operator	BDAL@DE
Sample Name	Sox_2	Instrument	micrOTOF-Q 10139
Comment			

Acquisition Parameter

Source Type	ESI	Ion Polarity	Positive	Set Nebulizer	0.4 Bar
Focus	Not active	Set Capillary	4500 V	Set Dry Heater	200 °C
Scan Begin	100 m/z	Set End Plate Offset	-500 V	Set Dry Gas	4.0 l/min
Scan End	2300 m/z	Set Collision Cell RF	600.0 Vpp	Set Divert Valve	Source

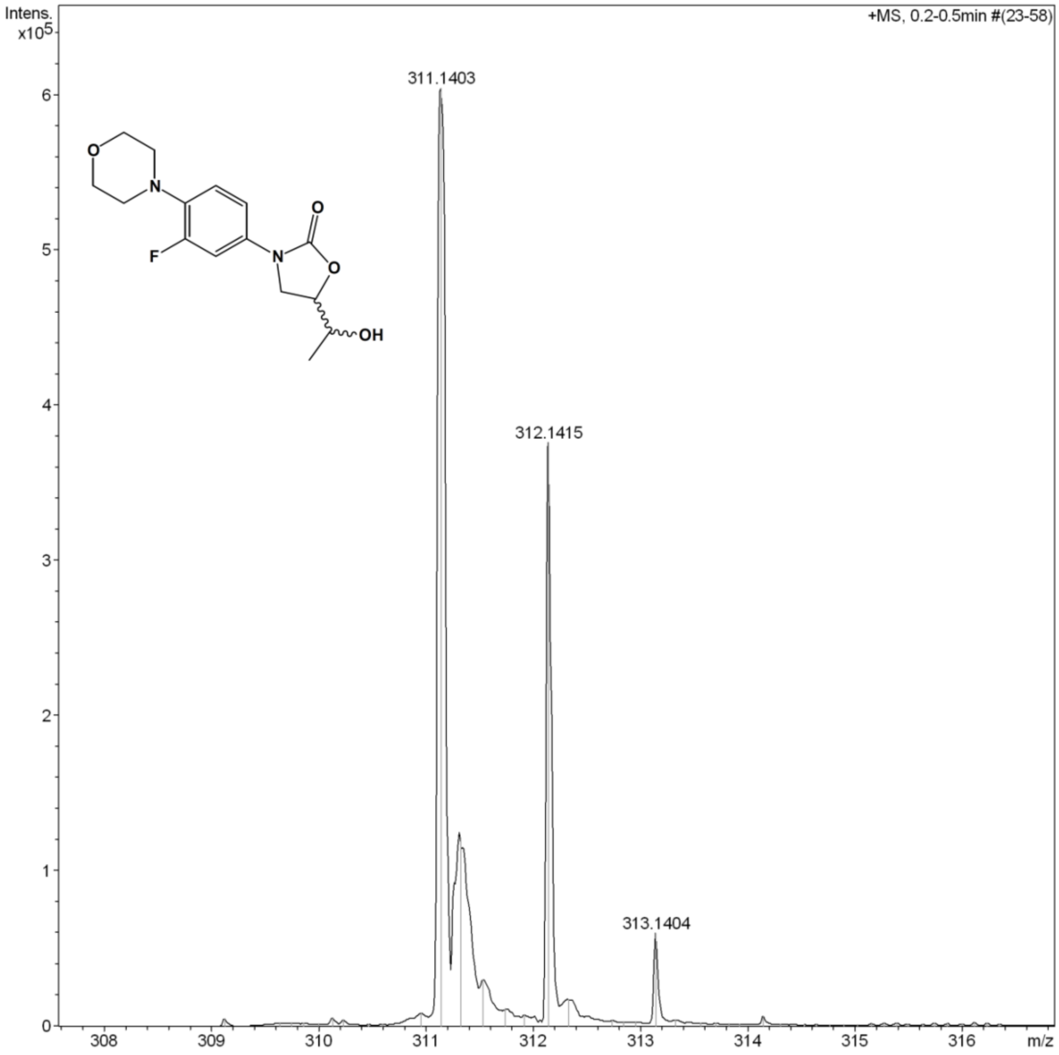

S20. HRMS of 3-(3-fluoro-4-morpholinophenyl)-5-(1-hydroxyethyl)oxazolidin-2-one (18b)

Display Report

Analysis Info

Analysis Name	D:\Data\YAHYA\Sox_3_83_01_7310.d	Acquisition Date	10/1/2015 10:57:59 AM
Method	fia.m	Operator	BDAL@DE
Sample Name	Sox_3	Instrument	micrOTOF-Q 10139
Comment			

Acquisition Parameter

Source Type	ESI	Ion Polarity	Positive	Set Nebulizer	0.4 Bar
Focus	Not active	Set Capillary	4500 V	Set Dry Heater	200 °C
Scan Begin	100 m/z	Set End Plate Offset	-500 V	Set Dry Gas	4.0 l/min
Scan End	2300 m/z	Set Collision Cell RF	600.0 Vpp	Set Divert Valve	Source

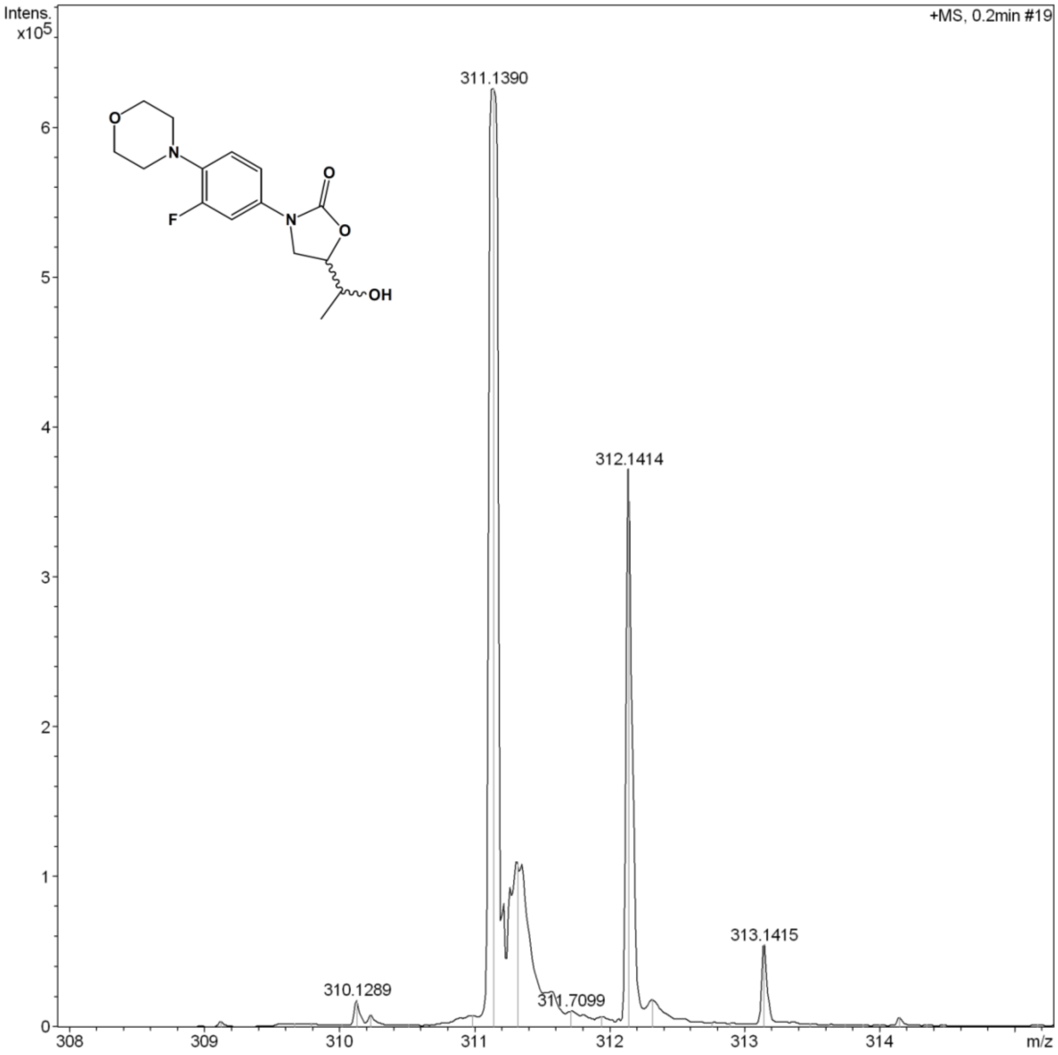

Supplement: Supplementary file 1 — Supporting Information [file OPEN-14-e202400432-s001.pdf]
